# Comparison of Serum Interleukin 6, Leptin, and Adiponectin Levels after Cesarean Section Under General Versus Spinal Anesthesia

**DOI:** 10.5812/aapm-162647

**Published:** 2025-08-20

**Authors:** Thaer Kareem Oleiwi Atabi, Ali Jabbari, Parvaneh Ebrahimzadeh, Hamzeh Bader Gazal, Somayeh Ghorbani Gholiabad, Ali Movafegh

**Affiliations:** 1 Department of Anesthesiology and Critical Care, Faculty of International , Golestan University of Medical Sciences, Gorgan, Iran; 2 Metabolic Disorders Research Center, Faculty of Medicine, Golestan University of Medical Sciences, Gorgan, Iran; 3 Department of Anesthesiology and Critical Care, Ischemic Disorders Research Center, Faculty of Medicine, Golestan University of Medical Sciences, Gorgan, Iran; 4 Department of Anesthesiology and Critical Care, Sayyad Shirazi Hospital, Golestan University of Medical Sciences, Gorgan, Iran; 5 Department of Anesthesia and Intensive Care, Al Zahra'a Teaching Hospital, Wasit Governorate, Kut, Iraq; 6 Cancer Research Center, Golestan University of Medical Sciences, Gorgan, Iran; 7 Department of Anesthesiology, Faculty of Medicine, Tehran University of Medical Sciences, Tehran, Iran

**Keywords:** Spinal Anesthesia, General Anesthesia, Cesarean Section, Inflammation, Cytokines, Adipokines

## Abstract

**Background:**

Inflammatory cytokines negatively impact postoperative reactions and outcomes in abdominal surgeries.

**Objectives:**

This study investigates the effect of general and spinal anesthesia (SA) on the serum levels of leptin, adiponectin, and interleukin-6 (IL-6) 24 hours after cesarean sections (CS) conducted with spinal or general anesthesia (GA).

**Methods:**

This cross-sectional study was conducted at Sayad Shirazi Hospital in Gorgan, IR. Iran, in 2024. Eighty pregnant women who were scheduled for CS were enrolled in the study. All participants satisfied the criteria for both general and SA, and the method of anesthesia was randomly assigned to each participant. Exclusion criteria included autoimmune diseases, preeclampsia, hypertension, gestational diabetes, a gestational age < 37 weeks, those who received blood transfusions and/or intubation. Participants were divided into two groups based on the anesthesia method after the CS: GA and SA. General anesthesia was administered using propofol and sodium thiopentone combined with atracurium and cis-atracurium. Bupivacaine and ropivacaine were used for SA. Peripheral blood samples were collected 24 hours post-operation to measure IL-6, leptin, and adiponectin levels.

**Results:**

A comparison of serum cytokine levels revealed that 24 hours after CS, IL-6, adiponectin, and leptin were significantly higher in the GA than in the SA group (P < 0.001 for all). In the SA group, there were positive and significant correlations among the following variables: IL-6 and leptin (r = 0.641, P < 0.001), IL-6 and adiponectin (r = 0.617, P < 0.001), as well as between leptin and adiponectin (r = 0.742, P < 0.001). In the GA group, a positive and significant correlation was found between the pro-inflammatory cytokines leptin and IL-6 (r = 0.316, P = 0.047).

**Conclusions:**

The present research found elevated leptin, IL-6, and adiponectin levels in CS cases that underwent GA.

## 1. Background

Gynecologists recommend cesarean sections (CS) as elective surgery to minimize risks for pregnant women and their fetuses (1). In the past two decades, the CS rate in Iran has risen significantly, exceeding the global average (2, 3). This increase has highlighted the importance of anesthesia, operating room conditions, and postoperative recovery for those undergoing the procedure. These factors must be thoroughly evaluated to provide the highest level of care and minimize potential complications related to CS (4, 5).

General anesthesia (GA) and spinal anesthesia (SA) are two widely used methods for expectant women to have a CS. In GA, the injection of anesthetic drugs temporarily halts neuromuscular activity. In contrast, in SA, injecting anesthetic medications into the spinal cord temporarily stops signal transmission to the central nervous system (6). Adverse effects that have been related to GA are typically caused by the drugs used in this anesthesia method. Some of these drugs, such as cis-atracurium and atracurium, have been shown to have different effects on blood pressure and alter the composition of white blood cells, including neutrophils and lymphocytes (7). Even in surgeries under SA, these changes have been associated with adverse postoperative outcomes such as nausea and vomiting (8). In the past decade, maternal complications associated with GA during CS have resulted in a notable decline in the application of this anesthesia method and an increase in the use of other anesthesia methods, especially SA. However, administering GA during CS is inevitable in some clinical situations (9). 

The immune system generally mediates anesthesia-related complications. The administration of anesthetic medications leads to varied immune system reactions, primarily resulting from shifts in immune cell populations. This includes alterations in natural killer cells, B and T lymphocytes, and macrophages, which in turn cause changes in cytokine secretion (10). One of the notable pro-inflammatory markers whose levels increase in proportion to post-surgical inflammation is interleukin-6 (IL-6) (11). A randomized clinical trial demonstrated significant IL-6 alteration after CS in ladies who were administered GA compared to those who received SA (12). It has also been shown that IL-6 has a parabolic trend of increasing and decreasing within 24 hours after abdominal surgeries with GA, independent of the type of drug injected. However, its levels can vary depending on the drug combination used (13).

The levels of cytokines secreted by adipose tissue (adipokines) undergo significant changes during pregnancy, and these cytokines play a notable role in pathological pregnancies (14). However, assessment of adipokines levels in post-cesarean section inflammation and their relationship with other inflammatory markers and anesthesia methods has received less attention (15). Leptin and adiponectin are two well-known adipokines that, in addition to their metabolic functions, play a key role in regulating immune responses. As a pro-inflammatory cytokine, Leptin promotes the multiplication of naive T cells while facilitating their differentiation into a Th1 immune response profile. Adiponectin is known for its anti-inflammatory effects, which may stem from its capacity to inhibit the production of interferon (IFN)-γ and tumor necrosis factor (TNF). This inhibition can result in a rise in the levels of various anti-inflammatory cytokines (16).

Disruption of anti-inflammatory and pro-inflammatory processes in abdominal surgery can be associated with adverse postoperative complications, including delayed recovery and various comorbidities (17). Appropriate anesthesia methods and the anesthesiologist's expertise in operating room management can influence immune cells' stress and inflammatory responses during and after the operation (18). 

## 2. Objectives

This study investigated the relationship between inflammatory mediators and SA and GA in expectant women 24 hours after CS.

## 3. Methods

### 3.1. Patient’s Inclusion and Methods of Anesthesia

In this cross-sectional study, pregnant women who were scheduled for elective CS were recruited from the outpatient antenatal clinic to participate in this study. All participants satisfied the criteria for both general and SA, and the method of anesthesia was randomly assigned to each participant. Parturients with autoimmune diseases, preeclampsia, hypertension, gestational diabetes, a gestational age of less than 37 weeks, those who received blood transfusions during CS, and those who underwent intubation were excluded from the study. After the CS, based on the anesthesia method administered, participants were divided into two groups: GA and SA.

All women were fasting (NPO) for at least 6 hours before anesthesia. Monitoring from the beginning of the anesthesia to the end of the CS included electrocardiography, non-invasive blood pressure, pulse oximetry, capnography (ETCO2), and BIS electroencephalogram monitoring.

General anesthesia was started without premedication. In the first step, an initial hypnotic drug, including 1.5 - 2.5 mg/kg propofol or 3 - 5 mg/kg sodium thiopental, was prescribed to sedate the pregnant women. Then, the patients were stabilized with sevoflurane/ isoflurane, and ventilation was performed following neuromuscular blocking by injection of 1 mg/kg succinylcholine and 0.2 - 0.3 mg/kg atracurium or cis-atracurium. After delivery, anesthesia was continued through IV injections of a combination of 2.5 mg midazolam and a narcotic, including 0.2 - 0.5 µg/kg fentanyl or 0.02 - 0.05 µg/kg sufentanil. In cases of hemodynamic instability, 0.3 mg/kg etomidate was administrated.

SA was administered using 10 - 12 mg of bupivacaine or ropivacaine. For this procedure, 2.5 - 3 ml of the medication was injected with a spinal needle (25-gauge) into the intervertebral space of the lumbar vertebrae (L3-L4 or L4-L5).

All subjects after CS used a patient-controlled analgesia pump containing 100 cc of nalbuphine 0.5 mg/mL to reduce postoperative pain.

Twenty-four hours after the operation was completed and the subjects were transferred to the general ward, they were chosen according to the inclusion criteria, with 40 women selected for each group. All participants signed an informed consent form based on the Helsinki Declaration Ethical Principles. A volume of 5 mL of vein blood was obtained from each participant. After centrifugation at 2700 RPM, the separated sera were transferred and held in a -70°C freezer until laboratory tests were conducted.

### 3.2. Laboratory Tests

Laboratory tests included two groups of biochemical and immunological tests. Biochemical tests included blood sugar, triglycerides, cholesterol, HDL, and LDL, performed by photometry. The ELISA method was also used to measure insulin levels (BETA Biomed, USA).

Immunological tests included leptin, adiponectin, and IL-6, which were performed using ELISA kits (ZellBio GmbH, Germany).

### 3.3. Statistical Analysis

The data analysis was conducted using SPSS software version 16. The Shapiro-Wilk test checked the data's normality. Depending on the data distribution, an independent *t*-test or a Mann-Whitney U-test was conducted to compare the two groups. The correlation between cytokines was evaluated using either the Pearson or Spearman correlation coefficient tests. Results were reported with a 95% confidence interval (CI 95%), and a P-value below 0.05 was deemed statistically significant.

## 4. Results

**Figure 1.**
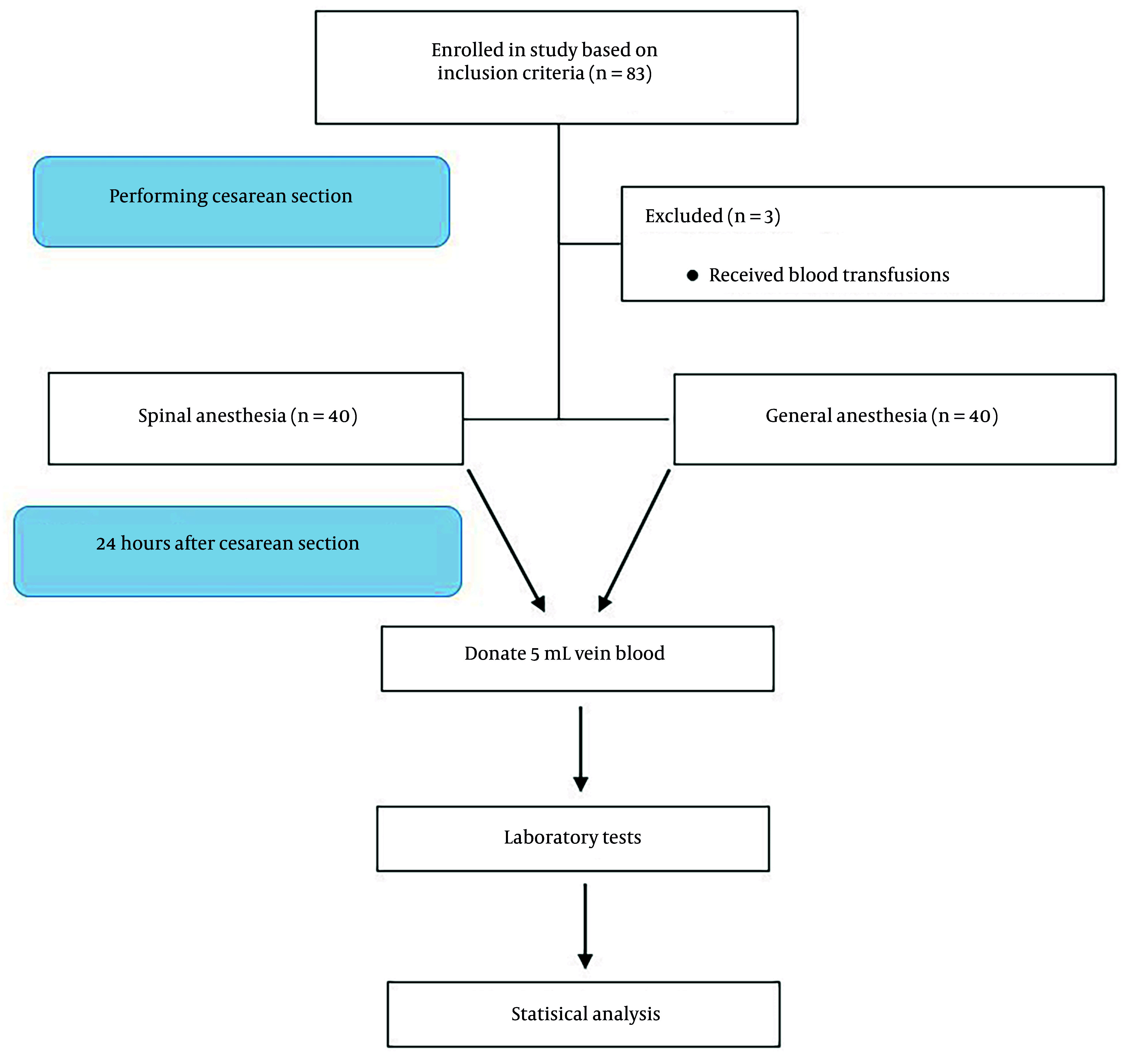
Flowchart of the study

Eighty parturients were included in this study after undergoing a cesarean section. Of these, 40 were assigned to the GA group and 40 were assigned to the SA group. Figure 1 provides additional information regarding the inclusion criteria and the stages of the study.

The ages of the participants in the two groups were comparable (P-value > 0.05). Furthermore, there was no notable difference in the length of CS between them (P-value > 0.05). The two groups had no significant differences in glycemia indices and lipid profile (P-value > 0.05) (Table 1). 

**Table 1.** Comparison of Demographic, Clinical, and Biochemical Indicators Between General and Spinal Anesthesia (N = 40) ^a, b^

Variables	GA	SA	P-Value
**Age (y) ** ^**c**^	29.75 ± 7.28	30.95 ± 7.90	0.428
**Duration of operation (min) ** ^**c**^	71.30 ± 10.52	70.74 ± 6.75	0.549
**FBS (mg/dL) ** ^**c**^	90.01 ± 9.75	95.46 ± 14.32	0.077
**Triglyceride (mg/dL) ** ^**c**^	130.07 ± 38.74	148.03 ± 46.17	0.063
**Total cholesterol (mg/dL) ** ^**d**^	190.77 ± 33.90	193.06 ± 42.87	0.704
**LDL-C (mg/dL) ** ^**d**^	112.47 ± 35.92	110.42 ± 46.19	0.866
**HDL-C (mg/dL) ** ^**c**^	52.28 ± 4.58	53.04 ± 5.42	0.502
**Insulin (µIU/mL) ** ^**d**^	9.59 ± 9.93	9.39 ± 7.93	0.885

Abbreviations: GA, general anesthesia; SA, spinal anesthesia; FBS, fasting blood sugar; HDL-C, high-density lipoprotein-cholesterol; LDL-C, low-density lipoprotein-cholesterol.

^a^ No difference was observed in demographic and clinical variables across groups

^b^ All values are expressed as mean ± standard deviation.

^c^ Independent *t*-test.

^d^ Mann-Whitney U-test.

A comparison of serum cytokine levels showed that after 24 hours of CS, IL-6, adiponectin, and leptin levels significantly increased in the GA compared to the SA (Table 2). 

**Table 2.** Comparison of IL-6, Adiponectin, and Leptin Between Two Methods of Anesthesia (N = 40) ^a, b^

Variables	GA	SA	P-Value
**IL-6 (pg/mL) ** ^**c**^	133.62 ± 15.51	109.84 ± 30.34	0.001
**Adiponectin (mg/L) ** ^**d**^	7.54 ± 0.95	5.15 ± 1.69	0.001
**Leptin (ng/mL) ** ^**d**^	215.08 ± 21.01	181.42 ± 41.93	0.001

Abbreviations: GA, general anesthesia; SA, spinal anesthesia; IL-6, Interleukin 6.

^a^ All values are presented as mean ± standard deviation.

^b^ Twenty-four hours after a CS, serum levels of leptin, adiponectin, and IL-6 were significantly higher in GA than in SA.

^c^ Independent *t*-test.

^d^ Mann-Whitney U-test.

**Figure 2.**
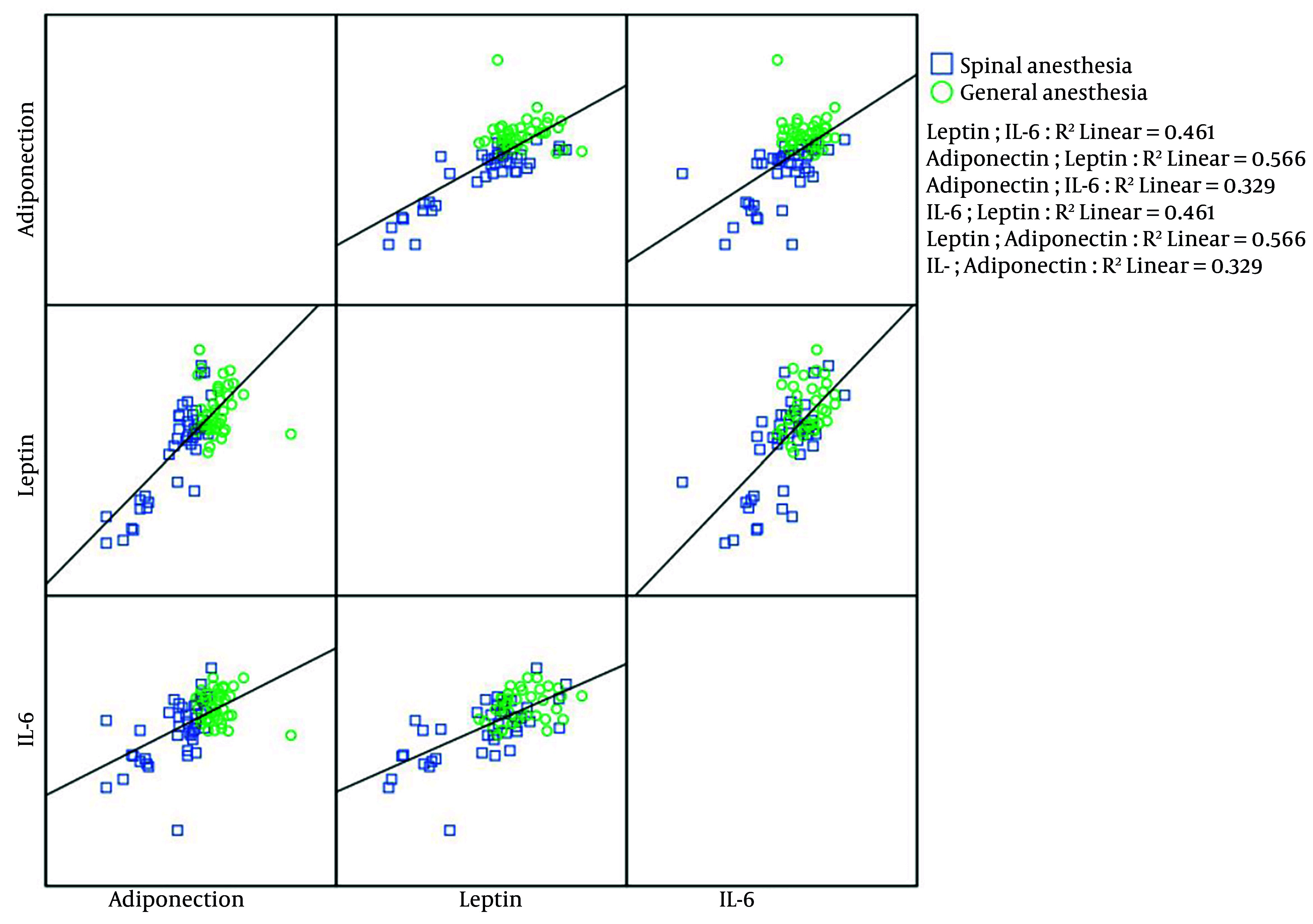
The scatterplot diagram investigated the correlation among interleukin-6 (IL-6), leptin, and adiponectin in the general and spinal anesthesia (SA) groups. The SA group exhibited positive and significant correlations among IL-6, leptin, and adiponectin. In contrast, in the general anesthesia (GA) group, a correlation was only found between IL-6 and leptin.

The bivariate correlations between cytokines are presented in Figure 2. In the SA group, Spearman's rank correlation showed that IL-6 positively and significantly correlated with leptin (r = 0.641, P-value < 0.001) and adiponectin (r = 0.617, P-value < 0.001) levels. In this group, leptin and adiponectin also had significant positive correlations (r = 0.742, P-value < 0.001). In the GA group, IL-6 and leptin were the only cytokines with a significant positive correlation, as exhibited by the Pearson coefficient (r = 0.316, P-value = 0.047).

## 5. Discussion

Our study explored the inflammatory status of pregnant women who underwent CS and were anesthetized using either spinal or general techniques, comparing the results 24 hours after the procedure. Our findings indicated that levels of inflammatory cytokines in the blood were significantly higher in the GA compared to the SA. The increase in cytokine concentrations during inflammatory responses is variable, but it has been shown that their increase peaks after 24 hours and remains high up to 72 hours (19). Our findings align with the results of Novac et al., who noted that serum IL-6 levels were significantly elevated in the GA compared to the SA, measured before, as well as 6 and 12 hours after the cesarean section (20). In another study, Vosoughian et al. observed no difference in preoperative and postoperative IL-6 serum levels in two groups of CS patients under general and SA. However, in the GA group, IL-6 levels increased after the operation (12). In another similar study, Hassanshahi et al. detected no significant relationship between IL-6 levels and anesthesia technique in elective CS (21). Although the drugs and doses used in our study and the mentioned studies were almost similar, the present study benefited from a larger sample size in both the GA and SA groups. The survey by Jafarzadeh et al. demonstrated that the expression of IL-6 at the mRNA level did not differ between the GA and SA techniques in CS (22). Another study has shown that there is no significant difference between the levels of IL-6, IL-10, and interleukin-1β (IL-1β) within 8 and 24 hours after inguinal hernia surgery under local, general, and SA (19). Our research findings indicate that the variations in serum cytokine levels after surgery are influenced not only by the method of anesthesia but also by other factors, such as the type of surgery performed and the patient’s condition prior to the procedure.

The results indicated that serum leptin levels increased in women undergoing CS with GA compared to SA. This study is the first to report leptin levels in CSs comparing general and SA methods. Increased serum leptin, a proinflammatory adipokine, has been associated with analgesic consumption after CS (23). Moreover, a study showed that leptin levels two hours after surgery were unrelated to preoperative carbohydrate intake (24). Our analysis also displayed a positive correlation between serum levels of leptin and IL-6 in both GA and SA groups. In a previous study, a positive correlation was observed between serum leptin and IL-6 levels, but this correlation was only observed in juveniles (25). Anesthesia methods, especially intravenous ones, affect the production and release of various pro-inflammatory and anti-inflammatory cytokines by altering the homeostasis of immune cells, thereby affecting the cytokine balance. Leptin released from peripheral blood monocytes and adipose tissue-resident macrophages directly stimulates the secretion of other proinflammatory cytokines, especially IL-6 (26).

Our study showed that serum levels of adiponectin, an anti-inflammatory adipokine, were significantly higher in the GA group than in the SA group. This study is the first to report adiponectin levels between GA and SA techniques in CS. Adiponectin is an anti-inflammatory cytokine, but prospective human studies have linked its increased levels with an increased mortality risk in people with various pathological conditions (27, 28). Previous studies have demonstrated a simultaneous increase in both proinflammatory and anti-inflammatory cytokines. For instance, Novak et al. found that 6 and 12 hours after CS, the serum levels of proinflammatory cytokines, including IL-6, tumor necrosis factor alpha (TNF-α), and interleukin-8, as well as anti-inflammatory cytokines, including interleukin-10 (IL-10) and interleukin-4 (IL-4), were significantly higher in parturients who underwent GA compared to those who received SA (20). As previously shown, the heightened secretion of adiponectin by macrophages serves as an essential stimulus for the expression and increase in serum levels of IL-10 (26). However, serum levels of IL-10 were not assessed in the present study. There were some limitations in this study, including the lack of measurement of serum levels of the cytokines before the operation. Another limitation of our study was the lack of measurement of the Body Mass Index (BMI) of pregnant women before and after CS. Although SA is associated with fewer complications than GA after cesarean section, including milder nausea and vomiting, the concurrent increase in the levels of this adipokine with two proinflammatory cytokines, IL-6, and leptin, requires further investigation.

### 5.1. Conclusions

In this study, we found that 24 hours after a cesarean section, the proinflammatory cytokines IL-6 and leptin, as well as the anti-inflammatory cytokine adiponectin, were higher in women who received GA compared to those who received SA. This suggests that SA may be more beneficial than GA for CS. To the best of our knowledge, this study is the first to examine the relationship between serum leptin and adiponectin levels with general or SA in CS. Further research is needed to explore the relationship between the serum levels of these cytokines and post-cesarean complications, including those affecting both mothers and newborns.

## Data Availability

The dataset presented in this study is available upon request from the corresponding author, either during submission or after publication. The data are not publicly available because they contain information that could compromise the privacy of research participants.
